# Commentary: Belatacept Does Not Inhibit Follicular T Cell-Dependent B-Cell Differentiation in Kidney Transplantation

**DOI:** 10.3389/fimmu.2017.01615

**Published:** 2017-11-23

**Authors:** Paul M. Schroder, Brian Ezekian, Mandy Ford, Stuart J. Knechtle, Jean Kwun

**Affiliations:** ^1^Duke Transplant Center, Department of Surgery, Duke University Medical Center, Durham, NC, United States; ^2^Emory Transplant Center, Department of Surgery, Emory University School of Medicine, Atlanta, GA, United States

**Keywords:** belatacept, costimulatory blockade, follicular helper T cells, secondary lymphoid organ, germinal center

Antibody-mediated rejection (AMR) of transplanted kidneys remains a challenging problem, despite contemporary improvements in immunosuppression. Therefore, identifying novel immunosuppressive agents and their effects on alloantibody production is important for improving outcomes. Alloantibody responses require B-cell activation and differentiation into antibody-producing plasma cells (PCs) with the help of T follicular helper (Tfh) cells. Disrupting this process could be key to developing more effective therapies for AMR. The recent study by de Graav et al. describes the effects of belatacept and tacrolimus on the process of Tfh cell–dependent B-cell differentiation (1). Their data provide important insights into the isolated effects of tacrolimus and belatacept *in vitro*, but do not demonstrate that belatacept fails to inhibit Tfh cell-dependent B-cell differentiation in kidney transplantation, as the article title suggests.

The first issue is that the study compared belatacept- and tacrolimus-treated renal transplant recipients, and as summarized in Table 2, found in 8 out of 11 measures of Tfh and B-cell activation and function examined, belatacept was as or more effective than tacrolimus. Since the study did not compare responses to unmodified immune responses in the absence of immunosuppression, it is likely an overstatement that belatacept does not inhibit Tfh-dependent B-cell differentiation. Rather, it appeared that in this study belatacept was not superior to tacrolimus in inhibiting Tfh and antibody responses.

One of the main limitations to immunologic studies involving human subjects is the restriction on sampling secondary lymphoid organs (SLOs) where the majority of Tfh cell–B-cell interactions occur (2). Therefore, peripheral blood is often used to evaluate the immunologic consequences of transplantation and immunosuppression. Unfortunately, as in the study by de Graav et al., this provides only a tiny snapshot of the immune system and generally fails to identify meaningful differences in cell populations or functions because the immune cells in circulation are largely not active participants in the alloimmune response. The only significant differences found between the belatacept and tacrolimus groups were after *in vitro* culturing with additional drug in the culture media. In addition, alloantibody was not measured directly from patient samples.

The authors acknowledge some of these limitations and cite prior studies that demonstrate an effect of belatacept on Tfh cell–B-cell interactions. The explanation for the lack of superior inhibition of T-cell–dependent B-cell differentiation in belatacept- versus tacrolimus-treated patients focuses on the facts that these studies fail to directly compare tacrolimus and belatacept because there are combination treatments that mask the true effect of belatacept alone. Prior studies have demonstrated that calcineurin inhibition with tacrolimus can have direct effects on B-cell proliferation and immunoglobulin production (3–5). The indirect effect of tacrolimus on B-cell immune responses through its disruption of T helper cell differentiation and function is also well documented (3, 6). It is also important to note that tacrolimus can dampen the effects of other immunosuppressive agents on B-cell immune responses when used in combination regimens (5). While no experimental model is perfect, in animal models where it is possible to track the Tfh and B-cell interactions within SLO, belatacept in non-human primates and CTLA4-Ig in mice have shown the ability to disrupt Tfh cells and prevent B-cell maturation to alloantibody-producing PCs. Kim et al. demonstrate that CTLA4-Ig alters the germinal center and reduces the population of Tfh cells in the spleens of skin-sensitized mice, leading to reduction in alloantibody production (7). Badell et al. also show inhibition of adoptively transferred donor-specific Tfh cells in the draining lymph nodes with CTLA4-Ig after skin transplantation in mice (8). Our study in non-human primates demonstrates that in a model of AMR using tacrolimus-based immunosuppression, subjects treated with belatacept showed reduced B-cell proliferation, number of CD4^+^PD1^+^ T-cells, and production of IL-21 within the lymph nodes compared with those without belatacept treatment (9). These results demonstrate the ability of belatacept to disrupt Tfh cell-mediated B-cell maturation in the context of alloimmune responses, as they measure these effects in the SLO where the Tfh cell–B-cell interactions occur and the drug is likely exerting its most potent effects, not in peripheral blood or in culture systems.

Correctly defining Tfh cells is another key aspect of understanding the results of the abovementioned studies. Tfh cells are conventionally described as CD4^+^ T-cells that express the CXC-chemokine receptor 5 (CXCR5) and therefore localize to the B-cell follicles within SLO. They are further characterized by cell surface phenotype with expression of inducible T-cell costimulator (ICOS) and programmed cell death protein 1 (PD-1). These cells also express the transcription factor Bcl-6 and produce the cytokine IL-21 (10). These bona fide Tfh cells are separate from the population of CD4^+^ CXCR5^+^ T-cells in peripheral blood, so-called circulating or blood memory Tfh cells, which have distinct subsets based on their expression of ICOS, PD-1, CC-chemokine receptor 7 (CCR7), CXCR3, and CCR6 (11, 12). These different subsets have different capacities to assist B-cells in the humoral immune response. The de Graav et al. study makes no distinction between these circulating CD4^+^CXCR5^+^ T-cells and bona fide Tfh cells. In addition, no attempt is made to identify alterations in the subsets of these circulating Tfh cells, which may also have explained the differential ability of the belatacept-treated cells to stimulate B-cell maturation compared to tacrolimus.

In summary, the findings by de Graav et al. are compelling in their novel comparison of tacrolimus- and belatacept-based immunosuppressive regimens in kidney transplant recipients. However, their conclusion and title overstate the claim that belatacept has no effect on Tfh cell-dependent B-cell differentiation. Also, as more studies measure circulating Tfh cells, it will be important to remember they are distinct from the bona fide Tfh cells that reside in the follicles of SLO, and a complete evaluation of circulating Tfh cell subsets may be needed to understand the true effects of immunosuppressants on this cell population.

## Author Contributions

PS, BE, MF, SK, and JK participated in writing the manuscript.

## Conflict of Interest Statement

The authors declare that the research was conducted in the absence of any commercial or financial relationships that could be construed as a potential conflict of interest.
